# Endoscopic application of novel, infection‐free, advanced hemostatic material: Its usefulness to upper gastrointestinal oozing

**DOI:** 10.1002/deo2.25

**Published:** 2021-08-22

**Authors:** Yuto Kubo, Satoru Kobayashi, Keiichi Yamamoto, Yoshie Nakagawa, Kotaro Yamashita, Takuro Saito, Koji Tanaka, Tomoki Makino, Kazuyoshi Yamamoto, Tsuyoshi Takahashi, Yukinori Kurokawa, Makoto Yamasaki, Hidetoshi Eguchi, Yuichiro Doki, Kiyokazu Nakajima

**Affiliations:** ^1^ Department of Next Generation Endoscopic Intervention (Project ENGINE) Graduate School of Medicine Center of Medical Innovation and Translational Research Osaka University Osaka Japan; ^2^ Department of Gastroenterological Surgery Graduate School of Medicine Osaka University Osaka Japan; ^3^ 3‐D Matrix Ltd. Tokyo Japan; ^4^ FUSO Pharmaceutical Industries Osaka Japan

**Keywords:** endoscopic submucosal dissection, endoscopic surgery, flexible endoscopy, hemostatic material, self‐assembling peptides

## Abstract

**Background:**

Self‐assembling peptides (TDMs) comprise synthetic amphipathic peptides that immediately react to changes in pH and/or inorganic salts to transform into a gelatinous state. The first generation of these peptides (TDM‐621) is currently used as a hemostatic agent in Europe. However, TDM‐621 exhibits slow gel‐formation and low retention capabilities on tissue surfaces. The second generation (TDM‐623) was therefore developed to encourage faster gel‐formation and better tissue‐sealing capabilities.

**Aim:**

The aim of this study was to verify the efficacy of TDM‐623 in terms of its hemostatic effect in endoscopic surgery.

**Materials and methods:**

Evaluation of the hemostatic effect in endoscopic surgery (animal study) was performed using eight porcine in spine position. Following systemic heparinization, we established a “bleeding model” by endoscopic grasping forceps on the anterior walls of the stomach and duodenum. In the hemostasis method, an endoscope with a distal hood was brought into contact with the bleeding point, and 1 ml TDM‐623 was applied to the wound. After TDM‐623 gelation, the endoscope was removed, and the acute hemostatic effect (after 2 min) was confirmed.

**Result:**

In the endoscopic bleeding model, 17 of the 23 cases (74%) showed complete hemostatic effects on the anterior wall of the stomach, and 18 of the 20 cases (80%) on the anterior wall of the duodenum, respectively. None of the applied gels were displaced from the anterior walls of the stomach and duodenum.

**Conclusion:**

The new self‐assembling peptide (TDM‐623) showed high hemostatic effects. TDM‐623 had potential usefulness for upper gastrointestinal endoscopic surgery.

## BACKGROUND

Several hemostatic methods have been used in flexible endoscopic surgery [1, 2]. The hemostatic materials used are primarily of biological origin because flexible endoscopy has a low level of maneuverability and narrow field of view, such that the methods available are not as abundant as for laparotomy. Although the safety associated with biological hemostatic material use has increased recently [3, 4, 5], the risk of infection has not been ruled out [6, 7, 8, 9, 10]. Therefore, peptide hemostatic materials, a type of products of nonbiological origin, have been attracting more attention.

Self‐assembling peptides (TDMs) are amphipathic peptides that undergo sol‐gel transformation in response to changes in pH and/or inorganic salts. Because TDMs are entirely chemically synthesized and are not associated with any risk of infection, their clinical application as a new class of hemostatic agents is expected to gain popularity [11, 12]. The first generation of these peptides (TDM‐621; 3‐D Matrix, Tokyo, Japan) is currently being used as a hemostatic agent in Europe [13, 14, 15, 16]. However, TDM‐621 has some shortcomings, such as somewhat slow gel formation and low retention on tissue surfaces.

Therefore, TDM‐623 was developed as the second‐generation self‐assembling peptide capable of accelerated gel formation and improved tissue retention. We previously reported the usefulness and increased performance of TDM‐623 relative to TDM‐621 via a preclinical open laparotomy study [17]. TDM‐623 is characterized by higher tissue retention and transparent gel formation [17]; thus, it might be useful in not only open surgery but also flexible endoscopic treatment. In this study, we verified the usefulness of TDM‐623 in endoscopic upper gastrointestinal surgery.

## AIM

The aim of the present study was to verify the usefulness of TDM‐623 in endoscopic upper gastrointestinal surgery.

### TDM‐623

**FIGURE 1:**
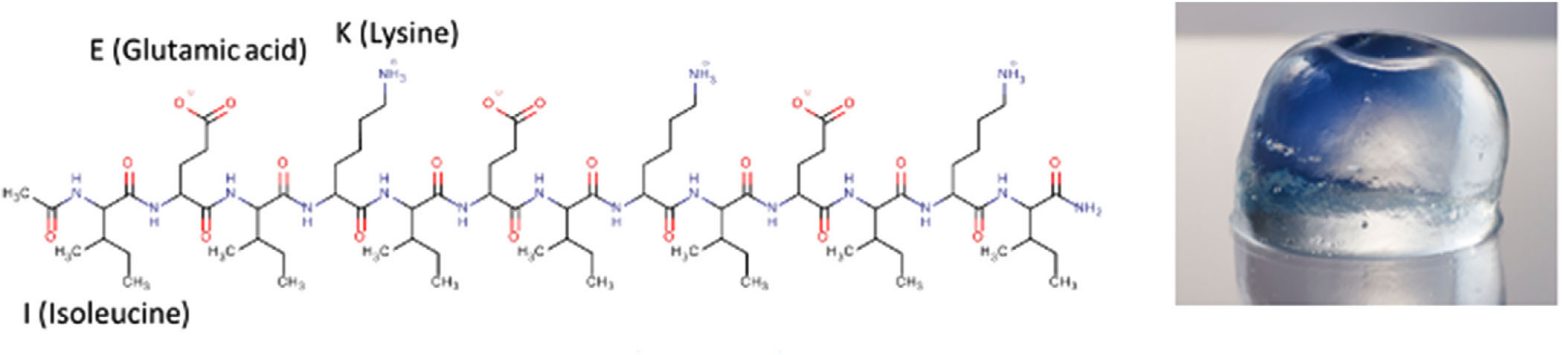
TDM‐623 is consisting of 14‐amino acid peptides that self‐assemble into nanofibers. The component of four repeats peptide of alternating hydrophilic natural amino acids (glutamic acid and lysine) and hydrophobic amino acids (isoleucine)

TDM‐623 is a newly developed material, consisting of 14‐amino acid peptides that self‐assemble into nanofibers (Figure 1). The component includes four repeats peptide of alternating hydrophilic natural amino acids (glutamic acid and lysine) and hydrophobic amino acids (isoleucine). Although TDM‐623 secures the basic hemostatic mechanism of TDM‐621, to address the aforementioned issues, TDM‐623 has recently been modified as an improved self‐assembling peptide with an expected high hemostatic effect, which accelerates gel‐formation speed and increases tissue retention than those of TDM‐621. It is considered that TDM‐623 is able to form a gel as soon as application to the bleeding site. Moreover, TDM‐621 requires storage in a cold place, but TDM‐623 can be stored at room temperature.

## METHODS

Eight female, 3‐month‐old mixed‐breed domestic swine, each weighing 35 kg, were used. The following experiments were performed under general anesthesia. The experiments were approved by the ethical committee of the Institutional Animal Care and Use Committee (approval number: IVT16‐25) and were conducted in accordance with the Declaration of Helsinki. All animals were euthanized at the end of each experiment using rapid intravenous administration (1 ml/kg) of saturated potassium chloride solution under deep anesthesia.

We intravenously injected low–molecular‐weight heparin (1 mg/kg) into eight porcine (weight, approximately 35 kg) and adjusted activated clotting time (ACT) ≥200. Then, under flexible endoscopy, we used endoscopic grasping forceps (FG‐47L‐1; Olympus Corporation, Tokyo, Japan) on the anterior wall of the upper gastrointestinal tract (the stomach and duodenum) to grasp the wall, thereby causing tissue injury and bleeding. The bleeding was evaluated based on time taken (in seconds) for the wound to be filled with blood. The obtained measurements were subsequently scored. To standardize bleeding, we modified the method proposed by George et al [18]. Originally, the degree of bleeding was scored using a five‐grade scale (Grades 0.5–4) by measuring the time necessary for each wound to fill with oozing blood. The present study modified this grading scale to a seven‐grade system by adding “Grade 0” (no bleeding) and “Grade 5” (spurting) (Table 1). This modification was performed because some punched wounds showed no oozing, whereas some showed arterial bleeding for which gel hemostasis was not indicated. Each wound was evaluated at its bleeding scale using the above‐mentioned system, and wounds between Grades 0.5 and 4 were included in the study. The exclusion criteria involved systemic low blood pressure (<80 mm Hg), Grade 0 and/or 5 bleeding.

**TABLE 1:** Bleeding was scored by measuring the time taken, in seconds, for the biopsy punch wound to fill with blood. Grade 0 (no exudative bleeding) and grade 5 (bleeding from a dissected blood vessel) were excluded from analyses

**Bleeding score**	**0**
Grade 0	No bleeding
Grade 0.5	Blood‐filled time to the wound over 4 s
Grade 1	3–4 s
Grade 2	2–3 s
Grade 3	1–2 s
Grade 4	Less than 1 s
Grade 5	Spurting bleeding

### Hemostatic effect under flexible endoscopy

**FIGURE 2:**
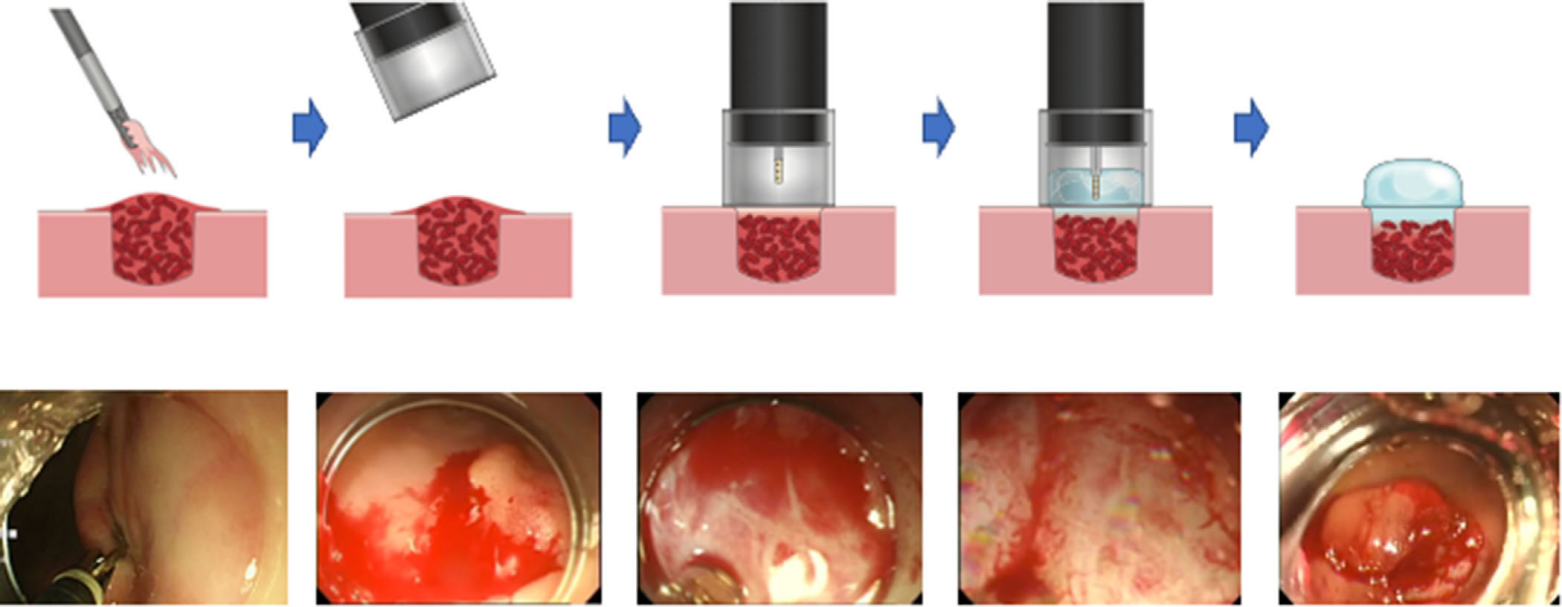
After creating a model bleeding lesion in the anterior wall of the stomach or the duodenum, a flexible endoscope with a transparent hood was brought into contact with the bleeding wound. Then, using a 5 ml syringe and a lavage catheter (end shower, ES‐120R; Yamashina Seiki Co. Ltd.), 1 ml of TDM‐623 was applied to the wound as it was filled in the hood. After 10 s, with the distal hood, the endoscope was slowly removed from the wound, and the tissue retention and complete hemostatic effects were verified at 2 and 5 min

We examined the stomach and duodenum of eight porcine using the experimental bleeding model described above. The following hemostasis procedure was verified by a total of five doctors. The procedure was performed under general anesthesia with the porcine in the supine position. After creating a model bleeding lesion in the anterior wall of the stomach or the duodenum, a flexible endoscope (GIF‐260J; Olympus Corporation) with a transparent hood (Elastic Touch, Slit & Hole type L; TOP Corporation, Tokyo, Japan) was brought into contact with the bleeding wound. Then, using a 5 ml syringe and a lavage catheter (End Shower, ES‐120R; Yamashina Seiki, Shiga Co. Ltd., Japan), 1 ml of TDM‐623 was applied to the wound as it was filled in the hood. After 10 s, with the distal hood, the endoscope was slowly removed from the wound, and the tissue retention and complete hemostatic effects were verified at 2 and 5 min (Supporting Information Video 1). Any substance displaced from the wound was considered to be bleeding (Figure 2). In this study, the hemostasis effect was judged whether gastric or duodenal mucosal bleeding was oozing out from the bottom of TDM‐623 after application of it.

(1) Evaluation of TDM in the stomach

The primary endpoint was hemostasis (whether or not complete hemostasis) at 2 min after application of TDM‐623. Secondary endpoints were verification of hemostatic effect 5 min after TDM administration, and usability of using under the flexible endoscopy.

(2) Evaluation of TDM in the duodenum

The evaluation of hemostatic effect in the duodenal hemorrhage model was performed in a same fashion of gastric experiments.

### Histological evaluation

Tissue samples were collected 6 h after the application of TDM‐623, and pathological specimens were prepared and stained with hematoxylin–eosin. We then evaluated how TDM‐623 filled into the dissected blood vessels and its effects on the surrounding tissue.

## RESULTS

(1) Evaluation of TDM in the stomach

**FIGURE 3:**
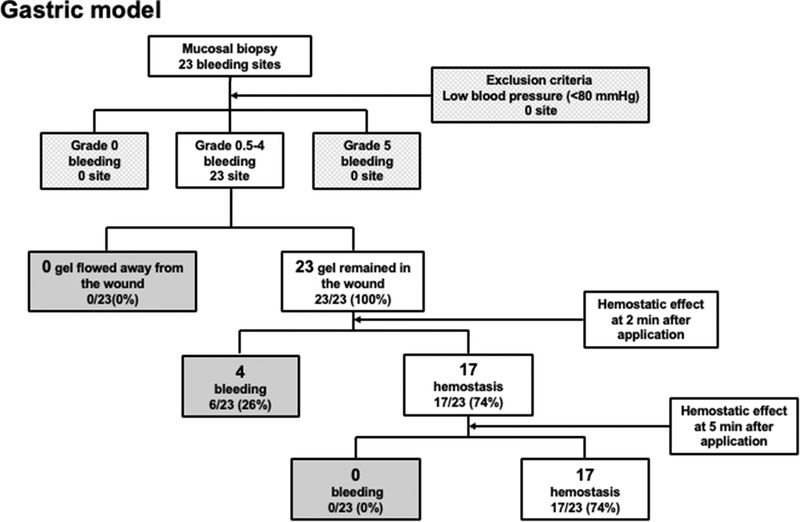
A total of 23 bleeding lesions were created in the anterior wall of the stomach. Complete hemostasis was observed in 17 of the 23 lesions (74%) after 2 min. After 5 min too, complete hemostasis was observed in 17 of the 23 sites (74%)

A total of 23 bleeding lesions were created in the anterior wall of the stomach. There were no adverse events by heparin administration, the median bleeding score was 4 (Table 2). A low blood pressure (<80 mm Hg), Grade 0 and/or 5 bleeding were none in this study. No lesions exhibited displacement of the gel from the wound after application. Complete hemostasis was observed in 17 of the 23 lesions (74%) after 2 min. After 5 min too, complete hemostasis was observed in 17 of the 23 sites (74%) (Figure 3).

(2) Evaluation of TDM in the duodenum

**TABLE 2:** A total of 23 bleeding lesions were created in the anterior wall of the stomach. The median bleeding score was 4. A total of 20 bleeding lesions were created in the anterior wall of the duodenum. The median bleeding score was 3

**Stomach**								
Bleeding score	0	0.5	1	2	3	4	5	Score (median)
Wound (*n*)	0	0	1	1	9	12	0	4
**Duodenum**								
Bleeding score	0	0.5	1	2	3	4	5	Score (median)
Wound (*n*)	0	0	0	6	11	3	0	3

**FIGURE 4:**
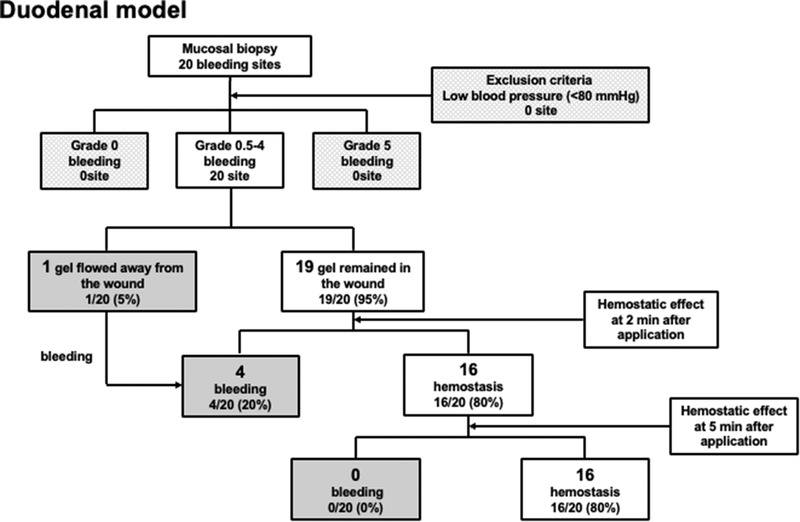
A total of 20 bleeding lesions were created in the anterior wall of the duodenum. Hemostatic effects were observed in 16 of the 20 lesions (80%) 2 min after application. Similarly, hemostatic effects were observed 5 min after application in 16 of the 20 lesions (80%)

A total of 20 bleeding lesions were created in the anterior wall of the duodenum, and there was no low blood pressure, Grade 0 and/or 5 bleeding during evaluation. The median bleeding score was 3 (Table 2), and only 1 of the 20 lesions exhibited displacement of the gel from the wound after application; the total success rate was 95% (19 of the 20 lesions), and hemostatic effects were confirmed in 16 of the 20 lesions (80%) 2 min after application. Similarly, hemostatic effects were confirmed 5 min after application in 16 of the 20 lesions (80%) (Figure 4).

TDM gel was transparent that did not interfere with the endoscopic visualization during and after application, thereby allowing easy confirmation of the hemostatic effects. In addition, because the gel could be applied to the bleeding lesion with pinpoint accuracy, fields of view other than the bleeding lesion were not interfered with.

In this study, the gel was applied using existing catheters without any problems; moreover, no trouble occurred with the use of the syringes.

### Histological evaluation of TDM‐623

**FIGURE 5:**
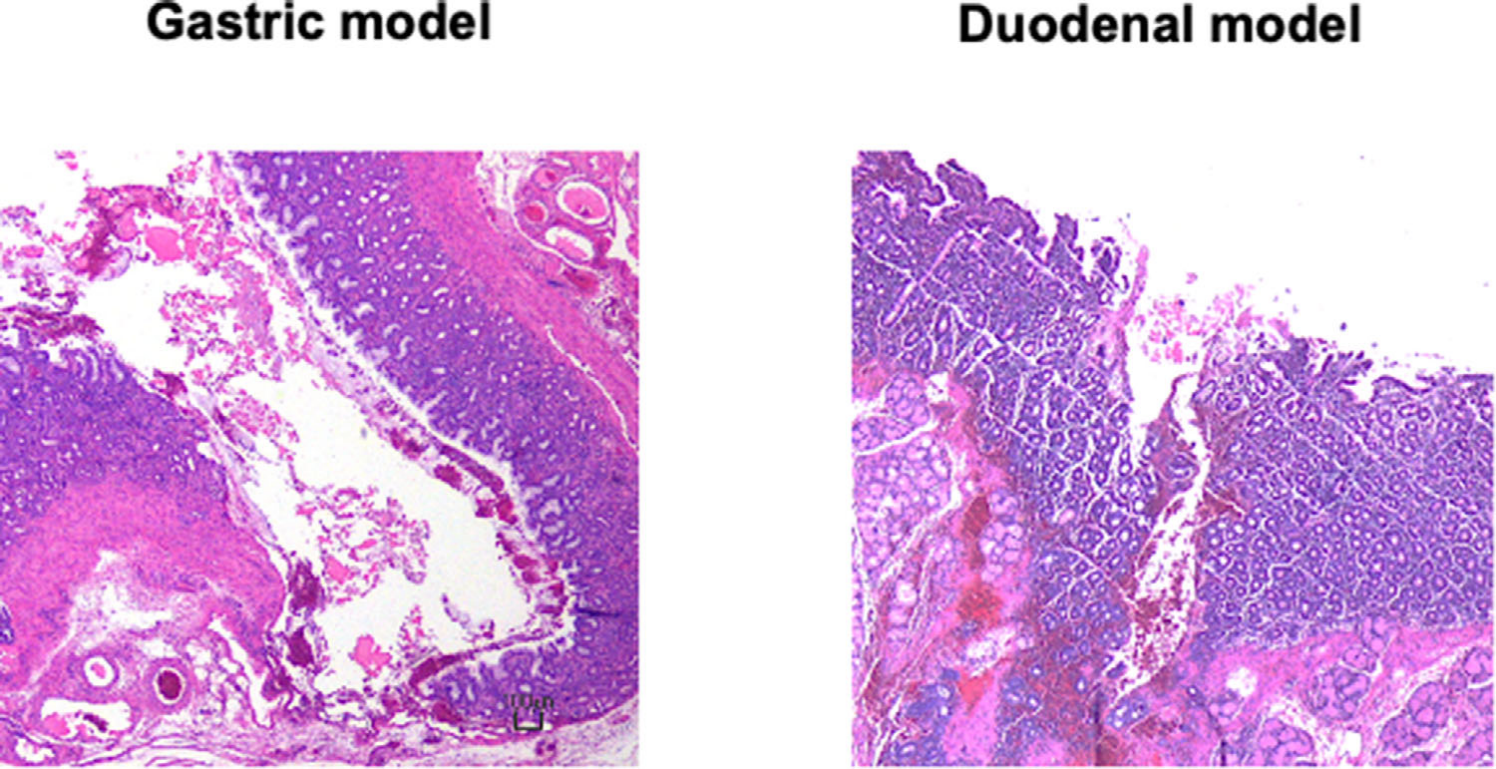
Although inflammatory cell infiltration was observed in the stomach and the duodenum owing to the filling and creation of wounds in these organs, no TDM‐induced severe inflammatory cell infiltration was observed

Although inflammatory cell infiltration was observed in the stomach and the duodenum due to traumatic reaction to the wounding, no TDM‐induced inflammatory cell infiltration was observed histologically (Figure 5).

## DISCUSSION

Because flexible endoscopes are equipped with only one or two working channels, it is difficult to perform precise hemostatic procedures while confirming bleeding points as such as laparotomy and laparoscopic surgery. In addition, flexible endoscopy is difficult to make fine hemostatic procedures, as open laparotomy or laparoscopic surgery; there is also a higher risk of intestinal perforation when devices with excessive electric energy are used for bleeding in a gastrointestinal wall [1, 2]. Therefore, a hemostatic agent is essential to support flexible endoscopy. Although the safety of biological hemostatic agents has improved [4], the associated risk of infection has not been fully ruled out [6, 7, 9]. Therefore, these nonbiological peptide hemostatic agents are attracting increasing attention [12, 13, 15, 16, 19].

TDM‐623 is a TDM‐621 analog with accelerated gel formation and higher tissue‐retention capability depending on the neutral pH range. Our previous study has suggested that the improved self‐assembling peptide TDM‐623 has greater hemostatic capability on the liver surface in heparinized bleeding than the first‐generation synthetic peptide TDM‐621, using large animal experiments [17]. However, this previous study was conducted under “open‐surgery” setting, and the hemostatic potential of TDM‐623 was not evaluated under endoscopic setting. The TDM‐based hemostatic procedure reported herein is an unprecedented method that takes advantage of the properties of the gel applied to the bleeding lesion after being filled in a transparent hood of an endoscope. This procedure allows the operator to observe the filling process of hemostatic gel in the wound, thereby enabling confirmation of the hemostatic effects. Also, our study considers that TDM‐623 can be applied with pinpoint accuracy to the bleeding site regardless of the ulcer size. Furthermore, because it is easy to apply the gel to oozing lesions, this procedure did not interfere with visualization. In case of failed hemostasis with TDM‐623, any other hemostatic methods can be applied, for example, endoscopic clip application, heat probe, or coagulation forceps, even without removing the gel due to its softness and transparency. In addition, the gel can be washed out from the field with water irrigation, if necessary. The hemostatic forceps have high risk of intestinal perforation when devices with excessive electric energy is used for bleeding in a gastrointestinal wall. In addition, inadequate clipping may lead to further bleeding. On the other hand, TDM‐623 has no risk of intestinal perforation and further bleeding. In terms of tissue retention and hemostatic effects, TDM‐623 demonstrates high levels of viscosity and gelation speed both in the stomach and duodenum. This explained why TDM‐623 shows a high tissue retention even in the anterior wall and high hemostatic effects at 74% in the gastric anterior wall and 80% in the duodenal anterior wall. Although histological evaluation revealed that the gel filled in the wound, no specific TDM‐induced inflammatory cell infiltration was observed.

A shortcoming of the TDM‐based hemostatic procedure is that the gel may stick to the hood after application. In this study, mild visual field disturbance occurred but had no noticeable negative impact on intraoperative procedures. In addition, using intraoperative endoscopic lavage, it was possible to sufficiently remove the gel stuck on the distal hood. A little force was required to press the syringe when applying the gel using a catheter; however, the gel could also be applied using existing commercially available catheters having no reported instrument‐related problems.

This study had several limitations that warrant discussion. First, it was a preclinical trial performed using large animals, and only eight porcine were used in this study. Second, the present study involved only five doctors. Third, only TDMs were evaluated and no comparison was made with no treatment spontaneous hemostasis, other hemostatic materials/devices, and procedure. Fourth, the bleeding models were created using grasping forceps, which resulted in uneven wound creation, and it was difficult to standardize the size of ulcers and scale of bleeding. Hence, we standardized not “bleeding” but “bleeding model” by using bleeding score. The endoscopic mucosal resection (EMR) and endoscopic submucosal dissection (ESD) need to dissect tissue under controlled hemostasis using coagulation forceps. Therefore, we consider that it is difficult to obtain standardize oozing using EMR and ESD. On the other hand, traumatizing tissue by biopsy forceps does not mean controlled hemostasis. Hence, we consider that traumatizing the mucosa to obtain adequate “oozing” by biopsy forceps is more appropriate, to create simple, suitable, reproducible bleeding models. Fifth, the hemostatic effects were examined after a relatively short time course of 5 min, delayed bleeding rate was not validated in our study. Moreover, it is unclear how long TDM‐623 gel is retained on the gastrointestinal wall. Hence, further studies need to consider the delayed bleeding rate and the duration of its retention in the gastric and duodenal walls. Finally, TDM application requires an endoscope with a distal hood and an application catheter as well as personnel who were trained to perform this application, which might not always be feasible.

## CONCLUSION

The new self‐assembling peptide TDM‐623 showed improved hemostatic effects even in flexible endoscopic surgery and demonstrated potential usefulness in the treatment of upper gastrointestinal bleeding.

## CONFLICT OF INTEREST

The authors declare that they have no conflict of interest.

## FUNDING INFORMATION

None.

## Supporting information


**Supplemental Video.1**: 1 ml of TDM‐623 was applied to the wound as it was filled in the hood, using a 5 ml syringe and a lavage catheter. After 10 s, with the distal hood, the endoscope was slowly removed from the wound.
